# Cryptanalysis of a Semi-Quantum Bi-Signature Scheme Based on W States

**DOI:** 10.3390/e24101408

**Published:** 2022-10-01

**Authors:** Chun-Wei Yang, Jason Lin, Chia-Wei Tsai, Ching-Lin Cheng

**Affiliations:** 1 Master Program for Digital Health Innovation, College of Humanities and Sciences, China Medical University, No. 100, Sec. 1, Jingmao Rd., Beitun Dist., Taichung 406040, Taiwan; 2 Department of Computer Science and Engineering, National Chung Hsing University, No. 145, Xingda Rd., South Dist., Taichung 40227, Taiwan; 3 Department of Computer Science and Information Engineering, National Taichung University of Science and Technology, No. 129, Sec. 3, Sanmin Rd., North Dist., Taichung 40401, Taiwan

**Keywords:** quantum cryptography, semi-quantum, quantum signature, bi-signature, W-like state

## Abstract

Recently, Zhao et al. proposed a semi-quantum bi-signature (SQBS) scheme based on W states with two quantum signers and just one classical verifier. In this study, we highlight three security issues with Zhao et al.’s SQBS scheme. In Zhao et al.’s SQBS protocol, an insider attacker can perform an impersonation attack in the verification phase and an impersonation attack in the signature phase to capture the private key. In addition, an eavesdropper can perform a man-in-the-middle attack to obtain all of the signer’s secret information. All of the above three attacks can pass the eavesdropping check. Without considering these security issues, the SQBS protocol could fail to ensure the signer’s secret information.

## 1. Introduction

With advances in quantum information science, various quantum techniques have been applied in quantum cryptography, such as entanglement swapping [1], quantum teleportation [2,3,4], and quantum remote control [5,6,7,8,9,10]. Quantum cryptography techniques can be used in various network communication environments, among which quantum signature is an important topic. In 2001, Gottesman and Chuang [11] proposed the first quantum signature concept. In 2002, Zeng and Keitel [12] proposed the first arbitrated quantum signature protocol based on the Green–Horne–Zeilinger (GHZ) state. Since then, various quantum signature protocols have been proposed, for example, arbitrated quantum signature [13,14,15,16,17,18,19,20], quantum blind signature [21,22,23,24,25,26], quantum proxy signature [27,28], and quantum group signature [29]. Inspired by the above quantum signature schemes [11,12,13,14,15,16,17,18,19,20,21,22,23,24,25,26,27,28,29], Zhao et al. [30] proposed a signature scheme based on the concept of “bi-signature.” In Zhao et al.’s quantum bi-signature protocol, two participants sign their signatures on the same message.

The aforementioned quantum signature schemes [11,12,13,14,15,16,17,18,19,20,21,22,23,24,25,26,27,28,29,30] all require the assumption that all participants in the protocol are quantum-capable, i.e., they must have devices such as photonic generators, quantum memory, and photonic measurement devices. In the absence of these devices, quantum signature protocols [11,12,13,14,15,16,17,18,19,20,21,22,23,24,25,26,27,28,29,30] cannot be executed. However, quantum devices are not widely available, and not all participants have access to such devices. In 2007, Boyer et al. [31] introduced the concept of a semi-quantum environment. In 2009, the same authors [32] proposed two types of semi-quantum key distribution protocols. Since then, various semi-quantum cryptographic protocols and applications have flourished. For example, various applications of the semi-quantum key distribution (SQKD) protocol have been reported. Some studies [33,34,35,36] have implemented SQKD protocols using single photons. Zhu et al. [37] design a SQKD protocol involving GHZ states. Considering the multiparticipant scenario, researchers have [38,39,40] proposed mediated SQKD protocols. Some studies [41] also discuss the implementation of SQKD protocols by excluding the measurement capabilities of classical participants. On the other hand, several studies [42,43,44,45] have investigated the implementation of authenticated SQKD protocols. Unlike SQKD protocols, another application environment is one in which the boss has quantum capabilities, and the agent has only classical capabilities. The boss divides the secret key into several parts and gives them to the agents for custody. The agents must work together to obtain the boss’s secret key. Current semi-quantum secret-sharing protocols are available in single photons [46,47,48,49], entangled states [50,51,52,53], GHZ states [54,55,56,57], W states [58], and cluster states [59]. In 2014, Zou and Qiu [60] proposed a three-step semi-quantum secure direct communication (SQSDC) protocol allowing a classical participant who does not have a quantum register to securely send a secret message to a quantum participant. Since then, many SQSDC protocols with an entanglement state [61,62,63,64,65,66,67,68] and without entanglement [69] have been proposed. Private comparison is primitive for many cryptographic tasks, and recently, several schemes for semi-quantum private comparison with single photons [70,71,72,73], Bell states [74,75,76,77], GHZ-like states [78,79,80], and W states [81] have been proposed.

In 2019, Zhao et al. [82] proposed a semi-quantum bi-signature (SQBS) scheme based on W-like states [83,84,85] and a quantum teleportation technique [86]. In the SQBS protocol, two participants are quantum-capable signers, and one is conventionally capable verifier. The main technique is to transmit the secret message of the signature to another signer through W-state teleportation technology. Then, the two signers transmit the signature messages to the verifier via their pre-shared keys. Finally, the verifier confirms that the two received signatures are identical, and the signature is completed.

Although Zhao et al. [82] proposed an SQBS protocol and proved the security of their protocol, in this study we highlight three security problems with the proposed SQBS protocol [82].

1. In the final step of the verification phase, the verifier (Charlie) performs an XOR operation with the pre-shared keys of two signers (Alice and Bob). If the verification passes, it means that the signature message is the same. Therefore, Bob can infer Alice’s pre-shared key and forge Alice’s signature later.
2. In the final step of the signature phase, the signer (Alice) transmits the signature message and the W-state measurement results to the verifier (Charlie) through the public classical channel. The public classical channel can be eavesdropped on and tampered with. Therefore, Bob can use the received secret message, Alice’s signature message, and measurement results to infer Alice’s pre-shared key, which can then be used to forge Alice’s signature.
3. The signer (Alice) transmits the secret message to another signer (Bob) through W-state teleportation technology; however, Alice and Bob do not perform any eavesdropping checks during the teleportation stage. Therefore, the eavesdropper (Eve) will be able to capture the secret message through a man-in-the-middle attack.

The rest of this paper is organized as follows. In Section 2, we review Zhao et al.’s SQBS protocol. In Section 3, we discuss three security issues associated with the protocol. Finally, in Section 4 we present our conclusions and discussion.

## 2. Review of Zhao et al.’s SQBS Protocol

**Figure 1:**
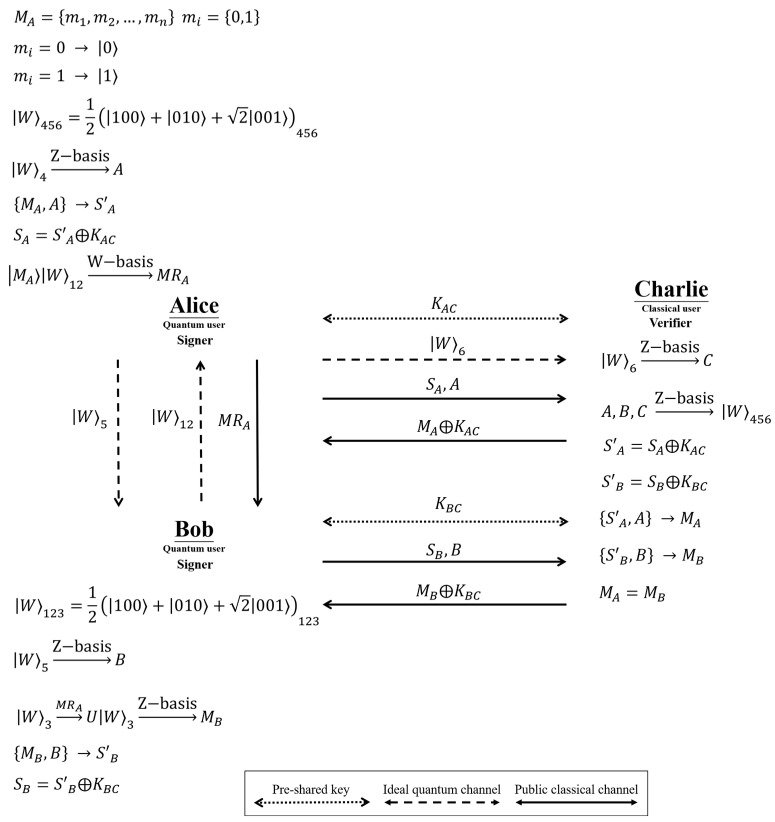
Zhao et al.’s SQBS protocol.

In Zhao et al.’s SQBS protocol [82], there are three participants: Alice and Bob (signers with quantum capabilities) and Charlie (a verifier with only classical capabilities); the classical capabilities of Charlie limit him to the use of the Z basis $\left\{\left|0\right.\rangle ,\left|1\right.\rangle \right\}$ to measure and generate single photons and to directly return the received quantum state. The eavesdropper, Eve, can perform any attack without violating the definition of quantum mechanics. Zhao et al.’s SQBS protocol is divided into three phases: the initial phase, the signature phase, and the verification phase. An overview of Zhao et al.’s SQBS protocol is shown in Figure 1. The detailed steps of Zhao et al.’s SQBS protocol are described as follows.

### 2.1. Initial Phase

In the initial phase, the pre-shared keys, $K_{AC}$ and $K_{BC}$, are allocated, and the W-like state is prepared to provide the execution requirements in the subsequent signature phase and verification phase.

 **Step 1.**  Alice prepares the secret message, $M_{A}=\left\{m_{1},m_{2},\ldots ,m_{n}\right\}$, where $m_{i}=\left\{0,1\right\}$. The agreed encoding rule is as follows: if the classical bit is “0”, then $\left|0\rangle \right.$ is generated; if the classical bit is “1”, then $\left|1\rangle \right.$ is generated.
 **Step 2.**  Bob and Alice prepare *n* sets of W-like states, ${\left|W\rangle \right.}_{123}=\frac{1}{2}{\left(\left|100\right.\rangle +\left|010\right.\rangle +\sqrt{2}\left|001\right.\rangle \right)}_{123}$ and ${\left|W\rangle \right.}_{456}=\frac{1}{2}{\left(\left|100\right.\rangle +\left|010\right.\rangle +\sqrt{2}\left|001\right.\rangle \right)}_{456}$.
 **Step 3.**  Through Krawec’s semi-quantum key distribution protocol [36], Alice and Charlie can share a private key, $K_{AC}$; Bob and Charlie can share a private key, $K_{BC}$.

### 2.2. Signature Phase

This phase focuses on Alice and Bob generating their respective signatures and sending them to Charlie. In addition, Alice sends the secret message, $M_{A}=\left\{m_{1},m_{2},\ldots ,m_{n}\right\}$, to Bob through the quantum teleportation of the W-like state.

 **Step 1.**  Alice sends ${\left|W\rangle \right.}_{5}$ and ${\left|W\rangle \right.}_{6}$ of ${\left|W\rangle \right.}_{456}=\frac{1}{2}{\left(\left|100\right.\rangle +\left|010\right.\rangle +\sqrt{2}\left|001\right.\rangle \right)}_{456}$ to Bob and Charlie, respectively, keeping ${\left|W\rangle \right.}_{4}$ for herself. Then, Bob sends ${\left|W\rangle \right.}_{12}$ of ${\left|W\rangle \right.}_{123}=\frac{1}{2}{\left(\left|100\right.\rangle +\left|010\right.\rangle +\sqrt{2}\left|001\right.\rangle \right)}_{123}$ to Alice and keeps ${\left|W\rangle \right.}_{3}$ for himself.
 **Step 2.**  Alice, Bob, and Charlie perform Z-basis measurements on their respective ${\left|W\rangle \right.}_{4}$, ${\left|W\rangle \right.}_{5}$, and ${\left|W\rangle \right.}_{6}$ and obtain the measurement results for *A*, *B*, and *C*.
 **Step 3.**  Based on Alice’s secret message ($M_{A}$) and the measurement result (*A*) of ${\left|W\rangle \right.}_{4}$, Alice’s signature message (${S^{\prime }}_{A}$) can be obtained through the coding rule listed in Table 1. Then, the signature message (${S^{\prime }}_{A}$) and the pre-shared key ($K_{AC}$) perform the exclusive or (XOR) operation to obtain Alice’s signature, $S_{A}={S^{\prime }}_{A}⨁K_{AC}$. Finally, Alice sends the signature ($S_{A}$) and the measurement result (*A*) to Charlie through the public classical channel.
 **Step 4.**  Alice generates the secret message ($M_{A}$) as a single photon $\left|M_{A}\right.\rangle$ according to the coding rules (i.e., if the classical bit is “0”, then generate $\left|0\rangle \right.$; if the classical bit is “1”, then $\left|1\rangle \right.$ is generated). Next, Alice measures $\left|M_{A}\right.\rangle$ with ${\left|W\rangle \right.}_{12}$ in the W-basis $\left\{{\left|\kappa \right.}^{\pm }\rangle =\frac{1}{2}\left(\left|010\rangle \right.+\left|001\rangle \right.\pm \sqrt{2}\left|100\rangle \right.\right),{\left|\gamma \right.}^{\pm }\rangle =\frac{1}{2}\left(\left|110\rangle \right.+\left|101\rangle \right.\pm \sqrt{2}\left|000\rangle \right.\right)\right\}$ and announces the measurement result to Bob. Based on the measurement result, Bob can perform the corresponding operation $\left\{\sigma_{1}=\left[\begin{array}{cc} 1 & 0\\ 0 & 1 \end{array}\right],\sigma_{2}=\left[\begin{array}{cc} 0 & 1\\ 1 & 0 \end{array}\right],\sigma_{3}=\left[\begin{array}{cc} 0 & -1\\ 1 & 0 \end{array}\right],\sigma_{4}=\left[\begin{array}{cc} 1 & 0\\ 0 & -1 \end{array}\right]\right\}$ in ${\left|W\rangle \right.}_{3}$ to obtain $\left|M_{B}\right.\rangle$. Finally, Bob measures $\left|M_{B}\right.\rangle$ in Z basis to obtain Alice’s secret message ($M_{B}$).
 **Step 5.**  Based on the received secret message ($M_{B}$) and the measurement result (*B*) of ${\left|W\rangle \right.}_{5}$, Bob can obtain the signature message (${S^{\prime }}_{B}$) via the coding rule listed in Table 1. Then, the signature message (${S^{\prime }}_{B}$) and the pre-shared key ($K_{BC}$) can be used to perform the XOR operation to obtain Bob’s signature, $S_{B}={S^{\prime }}_{B}⨁K_{BC}$. Finally, Bob sends the signature ($S_{B}$) and the measurement result (*B*) to Charlie through the public classical channel.

### 2.3. Verification Phase

This stage involves Charlie verifying whether Alice’s and Bob’s signatures are correct; the verification steps are as follows.

 **Step 1.**  Charlie first checks that Alice’s, Bob’s, and his own measurement results (*A*, *B*, and *C*) are consistent with the W-like state, ${\left|W\rangle \right.}_{456}=\frac{1}{2}{\left(\left|100\right.\rangle +\left|010\right.\rangle +\sqrt{2}\left|001\right.\rangle \right)}_{456}$. If the measurement result does not match, the SQBS protocol is canceled; otherwise, Charlie continues with the next step.
 **Step 2.**  Charlie can deduce Alice’s and Bob’s signature messages through $K_{AC}$ and $K_{BC}$ as ${S^{\prime }}_{A}=S_{A}⨁K_{AC}$ and ${S^{\prime }}_{B}=S_{B}⨁K_{BC}$, respectively.
 **Step 3.**  Charlie can deduce Alice’s secret message ($M_{A}$) by ${S^{\prime }}_{A}$ and *A* through the coding rules listed in Table 1. Similarly, Charlie can deduce Bob’s secret message ($M_{B}$) by ${S^{\prime }}_{B}$ and *B*.
 **Step 4.**  Charlie compares $M_{A}$ and $M_{B}$; if $M_{A}=M_{B}$, then Charlie accepts Alice’s and Bob’s signature; otherwise, Charlie rejects this signature. Finally, Charlie sends $M_{A}⨁K_{AC}$ and $M_{B}⨁K_{BC}$ to Alice and Bob, respectively.

## 3. Security Issues of Zhao et al.’s SQBS Protocol

In this study, we identified three security problems in Zhao et al.’s SQBS protocol: an impersonation attack in the verification phase, an impersonation attack in the signature phase, and a man-in-the-middle attack. The mechanisms of these three attack patterns are explained below.

### 3.1. Impersonation Attack in the Verification Phase

Consider Bob as the insider attacker. If Bob wants to impersonate Alice’s identity, he must obtain Alice’s private key with Charlie, $K_{AC}$. The following illustrates how Bob attacks.

In Step 4 of the verification phase, if Charlie accepts Alice’s and Bob’s signatures, then Charlie sends $M_{A}⨁K_{AC}$ and $M_{B}⨁K_{BC}$ to Alice and Bob, respectively. In this step, Bob intercepts the result of copying $M_{A}⨁K_{AC}$. Furthermore, if the signature is passed, it means that Bob’s message ($M_{B}$) is the same as $M_{A}$. Therefore, Bob can deduce that $M_{A}⨁K_{AC}=M_{B}⨁K_{AC}$ and learn the value of $K_{AC}$. In this way, Bob can impersonate Alice’s identity and communicate with Charlie through $K_{AC}$.

### 3.2. Impersonation Attack in the Signature Phase

Similarly, considering Bob as the insider attacker, if Bob wants to impersonate Alice’s identity, he must obtain Alice’s private key, $K_{AC}$. The following describes Bob’s attack strategy.

In Step 3 of the signature phase, Alice sends her signature ($S_{A}$) and the measurement result (*A*) to Charlie through the public classical channel. Hence, Bob can intercept and learn Alice’s signature ($S_{A}$) and the measurement result (*A*). In Step 4 of the signature phase, Bob can obtain Alice’s message ($M_{B}$) through the quantum teleportation of the W-like state. In this way, Bob has both $M_{B}$ and *A* and can deduce Alice’s ${S^{\prime }}_{A}$ in Table 1. Then, through ${S^{\prime }}_{A}$ and $S_{A}$, Bob can deduce Alice’s private key, $K_{AC}={S^{\prime }}_{A}⨁S_{A}$. Finally, Bob can impersonate Alice’s identity through $K_{AC}$ to communicate with Charlie.

### 3.3. Man-in-the-Middle Attack

**Figure 2:**
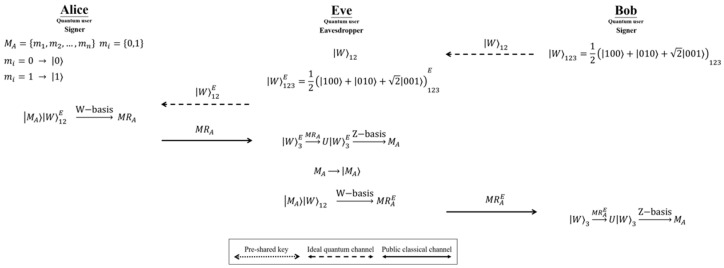
The process of the man-in-the-middle attack on Zhao’s SQBS protocol.

In the quantum signature protocol, the signer’s message cannot be known by anyone other than the signer. Therefore, Alice’s secret message ($M_{A}$) cannot be eavesdropped on. Once the signer’s secret message is leaked, the protocol is declared a failure. In Zhao’s SQBS protocol, the signers (Alice and Bob) protect Alice’s secret messages ($M_{A}$) through the quantum teleportation of the W-like state. However, in this study, we revealed that the eavesdropper, Eve, can perform a man-in-the-middle attack to obtain Alice’s secret message ($M_{A}$) without being detected. Because Alice and Bob do not have any protection or checking mechanism when executing quantum teleportation, Eve can capture the secret message ($M_{A}$). An overview of the man-in-the-middle attack on Zhao’s SQBS protocol is shown in Figure 2. The attack strategy is described as follows.

 **Step A1.**  In Step 1 of the signature phase, Bob sends the state ${\left|W\rangle \right.}_{12}$ of ${\left|W\rangle \right.}_{123}=\frac{1}{2}{\left(\left|100\right.\rangle +\left|010\right.\rangle +\sqrt{2}\left|001\right.\rangle \right)}_{123}$ to Alice. At this point, Eve intercepts ${\left|W\rangle \right.}_{12}$ and generates another set of W-like states, ${\left|W\rangle \right.}_{123}^{E}=\frac{1}{2}{\left(\left|100\right.\rangle +\left|010\right.\rangle +\sqrt{2}\left|001\right.\rangle \right)}_{123}^{E}$, and sends ${\left|W\rangle \right.}_{12}^{E}$ to Alice.
 **Step A2.**  In Step 4 of the signature phase, Alice measures $\left|M_{A}\right.\rangle$ and ${\left|W\rangle \right.}_{12}^{E}$ in the W-basis $\left\{{\left|\kappa \right.}^{\pm }\rangle =\frac{1}{2}\left(\left|010\rangle \right.+\left|001\rangle \right.\pm \sqrt{2}\left|100\rangle \right.\right),{\left|\gamma \right.}^{\pm }\rangle =\frac{1}{2}\left(\left|110\rangle \right.+\left|101\rangle \right.\pm \sqrt{2}\left|000\rangle \right.\right)\right\}$ and informs Bob of the measurement result. In this step, Eve intercepts Alice’s measurement result ($MR_{A}$); then, based on the measurement result ($MR_{A}$), Eve can perform the corresponding operation $\left\{\sigma_{1},\sigma_{2},\sigma_{3},\sigma_{4}\right\}$ in ${\left|W\rangle \right.}_{3}^{E}$ to obtain $\left|M_{A}\right.\rangle$. Thus, Eve can measure $\left|M_{A}\right.\rangle$ in the Z basis to obtain Alice’s secret message ($M_{A}$).
 **Step A3.**  After Eve obtains $M_{A}$, Eve generates $\left|M_{A}\right.\rangle$ and measures it with the intercepted ${\left|W\rangle \right.}_{12}$ in the W-basis $\left\{{\left|\kappa \right.}^{\pm }\rangle ,{\left|\gamma \right.}^{\pm }\rangle \right\}$. Then, Eve informs Bob of the measurement result ($MR_{A}^{E}$). Based on the measurement result ($MR_{A}^{E}$), Bob can perform the corresponding operation $\left\{\sigma_{1},\sigma_{2},\sigma_{3},\sigma_{4}\right\}$ in ${\left|W\rangle \right.}_{3}$ to obtain $\left|M_{A}\right.\rangle$. Finally, $\left|M_{A}\right.\rangle$ is measured through the Z-basis to obtain Alice’s secret message ($M_{A}$).

Because Eve’s attack does not destroy the secret message ($M_{A}$), Charlie’s inspection of Alice’s and Bob’s signatures will pass smoothly in the final verification stage. Therefore, Eve successfully executes the man-in-the-middle attack to capture Alice’s secret message ($M_{A}$) and is not discovered.

## 4. Conclusions

In this study, we highlight three security issues with Zhao et al.’s SQBS protocol: an impersonation attack in the verification phase, an impersonation attack in the signature phase, and a man-in-the-middle attack. In the impersonation attack, the insider attacker can capture the private key and impersonates the signer’s identity to communicate with the verifier. In the man-in-the-middle attack, the eavesdropper can obtain all the signer’s secret messages. All of the above three attacks can pass the eavesdropping check. Without considering these security issues, the SQBS protocol could fail to ensure the security of the signature. A possible solution is to add an eavesdropping check, for example, using decoy photons as an eavesdropping check. However, this requires an authenticated channel between the verifier and each signer and is therefore not very elegant. Improved solutions for this new issue in the SQBS protocol need to be designed in future research.

## Figures and Tables

**Table 1:** The coding rule of signature message.

	$M_{A}=0$	$M_{A}=1$
A=0	${S^{\prime }}_{A}=1$	${S^{\prime }}_{A}=0$
A=1	${S^{\prime }}_{A}=0$	${S^{\prime }}_{A}=1$

## Data Availability

Not applicable.

## References

[1] Xie C., Liu Y.-M., Chen J., Yin X., Zhang Z.-J. (2016). Quantum entanglement swapping of two arbitrary biqubit pure states. Sci. China Phys..

[2] Yuan H., Zhang Z. (2020). Optimizing the scheme of bidirectional controlled quantum teleportation with a genuine five-qubit entangled state. Mod. Phys. Lett. A.

[3] Zhang W., Li B., Zhang Z. (2020). Cyclic deterministic bidirectional quantum controlled teleportation with maximally seven-qubit entangled state. Laser Phys. Lett..

[4] Zhang Z., Xie C., Ye B. (2020). Teleportation with Mixing State from Two Bell States Due to Qubit Confusion. Int. J. Theor. Phys..

[5] Zhang Z., Xing H., Ye B., Xie C. (2020). Four-party quantum operation sharing with composite quantum channel in Bell and Yeo–Chua product state. Mod. Phys. Lett. B.

[6] Zhang Z., Zhang W., Ye B. (2020). Tripartite Quantum Operation Sharing with Six-Qubit Entangled State. Int. J. Theor. Phys..

[7] Zhang Z. (2021). Tripartite quantum operation sharing with six-qubit highly entangled state. Mod. Phys. Lett. A.

[8] Zhang Z., Yuan H. (2021). Deterministic tripartite sharing of an arbitrary single-qubit operation with the five-qubit cluster state in a given entanglement structure. Quantum Inf. Process..

[9] Zhang Z., Zhang L., Zhuge B., Ye B. (2021). Four-party deterministic quantum operation sharing with a generalized seven-qubit Brown state. Laser Phys. Lett..

[10] Zhang Z., Zhang L., Zhuge B., Yuan H., Ye B. (2021). Tripartite Quantum Operation Sharing with a Six-Qubit Absolutely Maximally Entangled State. Int. J. Theor. Phys..

[11] Gottesman D., Chuang I. (2001). Quantum digital signatures. arXiv.

[12] Zeng G., Keitel C.H. (2002). Arbitrated quantum-signature scheme. Phys. Rev. A.

[13] Lee H., Hong C., Kim H., Lim J., Yang H.J. (2004). Arbitrated quantum signature scheme with message recovery. Phys. Lett. A.

[14] Li Q., Chan W.H., Long D.Y. (2009). Arbitrated quantum signature scheme using Bell states. Phys. Rev. A.

[15] Dunjko V., Wallden P., Andersson E. (2014). Quantum digital signatures without quantum memory. Phys. Rev. Lett..

[16] Luo Y.P., Hwang T. (2014). Arbitrated quantum signature of classical messages without using authenticated classical channels. Quantum Inf. Process..

[17] Yang Y.G., Lei H., Liu Z.C., Zhou Y.H., Shi W.M. (2016). Arbitrated quantum signature scheme based on cluster states. Quantum Inf. Process..

[18] Chen F.L., Liu W.F., Chen S.G., Wang Z.H. (2017). Public-key quantum digital signature scheme with one-time pad private-key. Quantum Inf. Process..

[19] Zhang L., Sun H.W., Zhang K.J., Jia H.Y. (2017). An improved arbitrated quantum signature protocol based on the key-controlled chained CNOT encryption. Quantum Inf. Process..

[20] Xin X., Wang Z., He Q., Yang Q., Li F. (2019). New public-key quantum signature scheme with quantum one-way function. Int. J. Theor. Phys..

[21] Wen X., Niu X., Ji L., Tian Y. (2009). A weak blind signature scheme based on quantum cryptography. Opt. Commun..

[22] Su Q., Zheng H., Qiaoyan W., Wenmin L. (2010). Quantum blind signature based on two-state vector formalism. Opt. Commun..

[23] Yang C.W., Hwang T., Luo Y.P. (2013). Enhancement on “Quantum Blind Signature Based on Two-State Vector Formalism”. Quantum Inf. Process..

[24] Li W., Shi J., Shi R., Guo Y. (2017). Blind quantum signature with controlled four-particle cluster States. Int. J. Theor. Phys..

[25] Luo Y.P., Tsai S.L., Hwang T., Kao S.H. (2017). On “A new quantum blind signature with unlinkability”. Quantum Inf. Process..

[26] Guo X., Zhang J.Z., Xie S.C. (2018). A trusted third-party e-payment protocol based on quantum blind signature without entanglement. Int. J. Theor. Phys..

[27] Wang T.Y., Wei Z.L. (2012). One-time proxy signature based on quantum cryptography. Quantum Inf. Process..

[28] Yang C.W., Luo Y.P., Hwang T. (2014). Forgery attack on one-time proxy signature and the improvement. Quantum Inf. Process..

[29] Guo R., Cheng X. (2022). Cryptanalysis and improvement of a (t, n) threshold group signature scheme. Quantum Inf. Process..

[30] Zhao X.Q., Wang Y.Q., Gong L.H., Zeng Q.W. (2019). New bi-signature scheme based on GHZ states and W states. Int. J. Theor. Phys..

[31] Boyer M., Kenigsberg D., Mor T. (2007). Quantum key distribution with classical bob. Phys. Rev. Lett..

[32] Boyer M., Gelles R., Kenigsberg D., Mor T. (2009). Semiquantum key distribution. Phys. Rev. A.

[33] Zou X., Qiu D., Li L., Wu L., Li L. (2009). Semiquantum-key distribution using less than four quantum states. Phys. Rev. A.

[34] Krawec W.O. (2014). Restricted attacks on semi-quantum key distribution protocols. Quantum Inf. Process..

[35] Krawec W.O. (2016). Security of a semi-quantum protocol where reflections contribute to the secret key. Quantum Inf. Process..

[36] Wang M.M., Gong L.M., Shao L.H. (2019). Efficient semiquantum key distribution without entanglement. Quantum Inf. Process..

[37] Zhu K.N., Zhou N.R., Wang Y.Q., Wen X.J. (2018). Semi-quantum key distribution protocols with GHZ states. Int. J. Theor. Phys..

[38] Krawec W.O. (2015). Mediated semiquantum key distribution. Phys. Rev. A.

[39] Tsai C.W., Yang C.W., Lee N.Y. (2019). Lightweight mediated semi-quantum key distribution protocol. Mod. Phys. Lett. A.

[40] Tsai C.W., Yang C.W. (2021). Lightweight mediated semi-quantum key distribution protocol with a dishonest third party based on Bell states. Sci. Rep..

[41] Zou X., Qiu D., Zhang S., Mateus P. (2015). Semiquantum key distribution without invoking the classical party’s measurement capability. Quantum Inf. Process..

[42] Yu K.F., Yang C.W., Liao C.H., Hwang T. (2014). Authenticated semi-quantum key distribution protocol using Bell states. Quantum Inf. Process..

[43] Tsai C.W., Yang C.W. (2020). Lightweight authenticated semi-quantum key distribution protocol without trojan horse attack. Laser Phys. Lett..

[44] Wang H.W., Tsai C.W., Lin J., Huang Y.Y., Yang C.W. (2022). Efficient and secure measure-resend authenticated semi-quantum key distribution protocol against reflecting attack. Mathematics.

[45] Wang H.W., Tsai C.W., Lin J., Yang C.W. (2022). Authenticated semi-quantum key distribution protocol based on W states. Sensors.

[46] Gheorghiu V. (2012). Generalized semiquantum secret-sharing schemes. Phys. Rev. A.

[47] Yang C.W., Hwang T. (2013). Efficient key construction on semi-quantum secret sharing protocols. Int. J. Quantum Inf..

[48] Li Z., Li Q., Liu C., Peng Y., Chan W.H., Li L. (2018). Limited resource semiquantum secret sharing. Quantum Inf. Process..

[49] Tsai C.W., Chang Y.C., Lai Y.H., Yang C.W. (2020). Cryptanalysis of limited resource semi-quantum secret sharing. Quantum Inf. Process..

[50] Li Q., Chan W.H., Long D.Y. (2010). Semiquantum secret sharing using entangled states. Phys. Rev. A.

[51] Lin J., Yang C.W., Tsai C.W., Hwang T. (2013). Intercept-resend attacks on semiquantum secret sharing and the improvements. Int. J. Theor. Phys..

[52] Yin A.H., Tong Y. (2018). A novel semi-quantum secret sharing scheme using entangled states. Mod. Phys. Lett. B.

[53] Tian Y., Li J., Chen X.B., Ye C.Q., Li H.J. (2021). An efficient semi-quantum secret sharing protocol of specific bits. Quantum Inf. Process..

[54] Xie C., Li L., Qiu D. (2015). A novel semi-quantum secret sharing scheme of specific bits. Int. J. Theor. Phys..

[55] Yin A., Fu F. (2016). Eavesdropping on semi-quantum secret sharing scheme of specific bits. Int. J. Theor. Phys..

[56] Gao X., Zhang S., Chang Y. (2017). Cryptanalysis and improvement of the semi-quantum secret sharing protocol. Int. J. Theor. Phys..

[57] Tsai C.W., Yang C.W., Lin J. (2022). Multiparty mediated quantum secret sharing protocol. Quantum Inf. Process..

[58] Tsai C.W., Yang C.W., Lee N.Y. (2019). Semi-quantum secret sharing protocol using W-state. Mod. Phys. Lett. A.

[59] Li C., Ye C., Tian Y., Chen X.B., Li J. (2021). Cluster-state-based quantum secret sharing for users with different abilities. Quantum Inf. Process..

[60] Zou X., Qiu D. (2014). Three-step semiquantum secure direct communication protocol. Sci. China Phys. Mech..

[61] Zhang M.H., Li H.F., Xia Z.Q., Feng X.Y., Peng J.Y. (2017). Semiquantum secure direct communication using EPR pairs. Quantum Inf. Process..

[62] Xie C., Li L., Situ H., He J. (2018). Semi-quantum secure direct communication scheme based on Bell States. Int. J. Theor. Phys..

[63] Yan L., Sun Y., Chang Y., Zhang S., Wan G., Sheng Z. (2018). Semi-quantum protocol for deterministic secure quantum communication using Bell states. Quantum Inf. Process..

[64] Sun Y., Yan L., Chang Y., Zhang S., Shao T., Zhang Y. (2019). Two semi-quantum secure direct communication protocols based on Bell states. Mod. Phys. Lett. A.

[65] Yang C.W., Tsai C.W. (2019). Intercept-and-resend attack and improvement of semiquantum secure direct communication using EPR pairs. Quantum Inf. Process..

[66] Rong Z., Qiu D., Zou X. (2020). Semi-quantum secure direct communication using entanglement. Int. J. Theor. Phys..

[67] Yang C.W. (2020). Efficient and secure semi-quantum secure direct communication protocol against double CNOT attack. Quantum Inf. Process..

[68] Yang C.W., Tsai C.W. (2020). Advanced semi-quantum secure direct communication protocol based on bell states against flip attack. Quantum Inf. Process..

[69] Zhang X., Zhou R.G. (2022). An efficient and novel semi-quantum deterministic secure quantum communication protocol. Int. J. Theor. Phys..

[70] Yan-Feng L. (2018). Semi-quantum private comparison using single photons. Int. J. Theor. Phys..

[71] Ye T.Y., Ye C.Q. (2018). Measure-resend semi-quantum private comparison without entanglement. Int. J. Theor. Phys..

[72] Lin P.H., Hwang T., Tsai C.W. (2019). Efficient semi-quantum private comparison using single photons. Quantum Inf. Process..

[73] Li Y.C., Chen Z.Y., Xu Q.D., Gong L.H. (2022). Two semi-quantum private comparison protocols of size relation based on single particles. Int. J. Theor. Phys..

[74] Jiang L.Z. (2020). Semi-quantum private comparison based on Bell states. Quantum Inf. Process..

[75] Tsai C.W., Lin J., Yang C.W. (2021). Cryptanalysis and improvement in semi-quantum private comparison based on Bell states. Quantum Inf. Process..

[76] Xie L., Li Q., Yu F., Lou X., Zhang C. (2021). Cryptanalysis and improvement of a semi-quantum private comparison protocol based on Bell states. Quantum Inf. Process..

[77] Li Z., Liu T., Zhu H. (2022). Private comparison protocol for multiple semi-quantum users based on Bell States. Int. J. Theor. Phys..

[78] Tian Y., Li J., Chen X.B., Ye C.Q., Li C.Y., Hou Y.Y. (2021). An efficient semi-quantum private comparison without pre-shared keys. Quantum Inf. Process..

[79] Yan L., Zhang S., Chang Y., Wan G., Yang F. (2021). Semi-quantum private comparison protocol with three-particle G-like states. Quantum Inf. Process..

[80] Li Q., Li P., Xie L., Chen L., Quan J. (2022). Security analysis and improvement of a semi-quantum private comparison protocol with three-particle G-like states. Quantum Inf. Process..

[81] Tian Y., Li J., Ye C., Chen X.B., Li C. (2022). W-state-based semi-quantum private comparison. Int. J. Theor. Phys..

[82] Zhao X.Q., Chen H.Y., Wang Y.Q., Zhou N.R. (2019). Semi-quantum bi-signature scheme based on W states. Int. J. Theor. Phys..

[83] Ozaydin F., Bugu S., Yesilyurt C., Altintas A.A., Tame M., Özdemir Ş.K. (2014). Fusing multiple W states simultaneously with a Fredkin gate. Phys. Rev. A.

[84] Bugu S., Ozaydin F., Ferrus T., Kodera T. (2020). Preparing Multipartite Entangled Spin Qubits via Pauli Spin Blockade. Sci. Rep..

[85] Ozaydin F., Yesilyurt C., Bugu S., Koashi M. (2021). Deterministic preparation of W states via spin-photon interactions. Phys. Rev. A.

[86] Li K., Kong F.-Z., Yang M., Ozaydin F., Yang Q., Cao Z.-L. (2016). Generating multi-photon W-like states for perfect quantum teleportation and superdense coding. Quantum Inf. Process..

